# Thymolipoma-associated Myasthenia Gravis with High Titer of Anti-MuSKAb: A Case Report

**DOI:** 10.22088/IJMCM.BUMS.8.1.90

**Published:** 2016-06-28

**Authors:** Maryam Poursadeghfard, Amin Abolhasani Foroughi, Sina Karamimagham

**Affiliations:** 1 *Clinical Neurology Research Center, Department of Neurology, School of Medicine, Shiraz University of Medical Sciences, Shiraz, Iran.*; 2 *Medical Imaging Research Center, Department of Radiology, Shiraz University of Medical Sciences, Shiraz, Iran.*; 3 *Department of Pediatric Medicine, * *Shiraz University of Medical Sciences, Shiraz, Iran.*

**Keywords:** Myasthenia gravis, thymolipoma, anti-acetylcholine receptor antibody, anti-Muscle Specific Tyrosine Kinase- Antibody

## Abstract

Myasthenia gravis (MG) is a neuromuscular junction disorder caused by pathogenic autoantibodies to some parts of the post-synaptic muscle endplates. About 85% of generalized MG patients have autoantibodies against post-synaptic acetyl-choline receptors (AChR). From the 10-15% of the remaining patients, 45-50% are positive for Muscle Specific Tyrosine Kinase-Antibody (MuSK-Ab). It is believed that the thymus has a critical role in the pathogenesis of the disease with AChR-Ab, specially in patients with thymic abnormalities. In contrast, the role of thymus gland in MG with anti-MuSK-Ab is not clearly obvious. Patients with this antibody virtually have normal or only minimal follicular hyperplastic thymus. The presence of anti-MuSK Ab in a thymolipomatous (an uncommon tumor of thymus) MG is an atypical and new finding of MG because of not only thymolipoma but the presence of anti-MuSK antibodies which makes this case different from the previous reports of the antibodies-associated MG. Here, we present a young woman with thymolipoma and MG (a very uncommon kind of tumor-associated MG) and high level of anti-MuSK-Ab.

 Myasthenia Gravis (MG) is a neuromuscular junction disorder caused by pathogenic autoantibodies to some parts of the post-synaptic muscle endplates (1). It presents with fluctuating weakness in the striated muscles, but not all muscles are involved with the same severity and frequency. The distribution of the involvement from the most to the least is usually ocular, bulbar, proximal of the limbs and neck and sometimes respiratory muscles. Worsening of the weakness with sustained physical activity is a clue for diagnosis of MG (2).

About 85% of generalized MG patients have autoantibodies against post-synaptic acetyl-choline receptors (AChR). From the 10-15% of the remaining patients, 45-50% are positive for Muscle Specific Tyrosine Kinase-Antibody (MuSK-Ab) (3). These two different types of autoantibodies make a clear differentiation of MG sub-types.

It is believed that the thymus has a critical role in the pathogenesis of MG with AChR-Ab, specially in patients with the thymus abnormality (4). Some reports indicated that 100% of MG-associated thymoma have detectable AChR-Ab (5). Furthermore, no detectable MuSK antibody was reported in MG patients with thymoma (6).

Here, we present a young woman with thymolipoma and MG (a very uncommon kind of tumor-associated MG) and high level of anti-MuSK-Ab.

## Case presentation

The patient was a 24 year-old woman that was presented with ptosis, dysphagia, dyspnea and generalized weakness during the 3 previous weeks for the first time in June 2012. Past medical, family and drug histories were completely normal. She has no history of previous trauma or recent vaccination. Because of the worsening of dyspnea the day before, she was admitted in Nemazee Hospital, a medical academic center affiliated to Shiraz University of Medical Sciences, Shiraz, South of Iran.

Neurological examination revealed mild respiratory distress, unilateral ptosis, bilateral mild facial weakness, nasal speech after few seconds’ repetition of words, and generalized proximal muscle weakness.

**Fig. 1:**
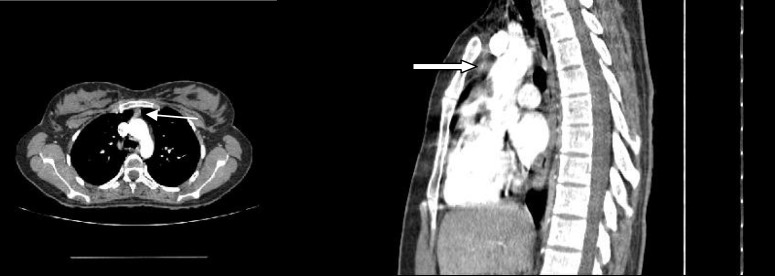
**Axial CT scan with sagittal reconstruction from mediastinum in a 24 years old lady.** A round centrally enhancing mass lesion in anterior mediastinum measuring 7.7 mm in transverse and 6 mm in anteroposterior diameter (white arrows). The images are taken with BrightSpeed GE 16 slice CT scanner, TI: 791 ms, kV: 120, mAs: 4, WL: 40, WW: 400, Slice thickness: 1.25mm

In paraclinic evaluation, tensilon (edropho-nium chloride) test for ptosis and nasal speech was positive. Repetitive nerve stimulation (3-5 Hz) performed on the trapezius and orbicularis oculi (in involved side) muscles showed 27% and 18% decremented pattern at rest. Complete blood count (CBC), blood sugar, biochemistry, liver and thyroid and renal function tests were all normal. Spiral chest CT scan was done for thymus evaluation which represented a small anterior mediastinal mass (Figure 1).

With MG diagnosis, Intravenous immunogl-obulin (IVIG) was started. She responded well to medication, and was discharged from hospital after completing the hospital treatment with oral prednisolone and azathioprine in a good state. Pathologic report of follow-up thymectomy showed thymolipoma, and documented the diagnosis of mediastinal mass detected in her chest CT scan.

6 months later, after starting to taper prednisolone, she developed repeated attack of dyspnea and dysphagia. In the second hospital admission during the disease relapse, AChR- Ab and anti-MuSK-Ab were requested. The titer of the AChR-Ab was 0.28 U/mL (<0.45 negative) and anti-MuSK-Ab 8.8 U/mL (<0.4 negative). 

## Discussion

Thymolipoma is a rare and slow-growing benign tumor of the anterior mediastinum and comprises 2-9% of all thymus neoplasms. Sometimes, it is related to systemic diseases such as Grave’ disease, aplastic anemia or other autoimmune disorders. Furthermore, MG is an autoimmune disease in which intra-thymus gland changes could be seen. In a rare medical condition, it is associated with thymolipoma while there are few case reports about the thymolipoma-associated MG (7-10).

Thymoma is another more common thymus neoplasm accompanied MG and has a close relationship to production of the AChR-Ab. On the other hand, almost all patients with MG and thymoma have a high serum level of AChR-Ab (11). In contrast, the role of the thymus gland in MG with the anti-MuSK-Ab is not clearly obvious. MuSK is an enzyme which supports post- synaptic membrane, and causes clustering of the AChR during neuromuscular junction development (12). Patients with this antibody virtually have normal or only minimal follicular hyperplastic thymus (13).

Our patient had thymolipoma and the major interesting point of her disease was high titer of the anti-MuSK-Ab with negative AChR-Ab.

As mentioned earlier, MuSK-associated MG has no or only minor thymus change and the role of the thymectomy as a treatment strategy is still unclear (13). When we reviewed the literature, we found no reports about the thymolipoma-associated MG with high titer of the anti-MuSK-Ab.

In our patient, both AChR and anti-MuSK antibodies were checked during repeated attack and second hospital admission. But, it had been performed after thymectomy and immunos-uppressant therapy (prednisolone and azathioprine), and it might affect the real result of the autoantibodies assessment. The effect of thymec-tomy on the AChR-Ab titer is still controversial. Some data have shown a decrease, while others reported an increase in its titer (14). Actually, it is not clear whether the patient was a thymolipoma-associated MG who had both antibodies simultaneously or had only anti-MuSK Ab. In 2004, Ohta et al. showed an association of the anti-AChR and anti-MuSK antibodies with MG. They provided a recombinant protein of the extracellular domain of human MuSK which was a new specific and sensitive rout to detect the presence of MuSK antibody in MG. Standard tests for MUSK antibodies use radioimmunopre-cipitation or ELISA (1). In spite of the previous data, they found that 10.5% of AChR-Ab positive patients (with or without thymoma) were also MuSK-Ab positive (6). Nevertheless, the presence of anti-MuSK-Ab in a thymolipomatous MG (whit or without AChR-Ab) is an atypical and new finding of MG which makes this case different from the previous reports of the antibodies-associated MG.

## References

[1] Gilhus NE, Verschuuren JJ (2015). Myasthenia gravis: subgroup classification and therapeutic strategies. Lancet Neurol.

[2] Jayam Trouth A, Dabi A, Solieman N (2012). Myasthenia gravis: a review. Autoimmune Dis.

[3] Diaz-Manera J, Rojas-Garcia R, Gallardo E (2007). Antibodies to AChR, MuSK and VGKC in a patient with myasthenia gravis and Morvan's syndrome. Nat Clin Pract Neurol.

[4] Meriggioli MN, Sanders DB (2012). Muscle autoantibodies in myasthenia gravis: beyond diagnosis?. Expert Rev Clin Immunol.

[5] Aarli J, Lisak Rp (1994). Myasthenia gravis and thymoma. Handbook of Myasthenia Gravis and Myasthenic Syndromes.

[6] Ohta K, Shigemoto K, Kubo S (2004). MuSK antibodies in AChR Ab-seropositive MG vs AChR Ab-seronegative MG. Neurology.

[7] Kawai H, Orino K, Minamiya Y (2005). [Thymolipoma in association with myasthenia gravis; report of a case]. Kyobu Geka.

[8] Takamori S, Hayashi A, Tayama K (1997). Thymolipoma associated with myasthenia gravis. SCJ.

[9] Taniguchi T, Usami N, Oohata N (2009). [Thymolipoma associated with myasthenia gravis]. Kyobu Geka.

[10] Tsukioka T, Inoue K, Iwata T (2007). Thymolipoma associated with myasthenia gravis. Gen Thorac Cardiovasc Surg.

[11] Nakajima J, Murakawa T, Fukami T (2008). Postthymectomy Myasthenia Gravis: Relationship With Thymoma and Antiacetylcholine Receptor Antibody. Ann Thorac Surg.

[12] El-Salem K, Yassin A, Al-Hayk K (2014). Treatment of MuSK-Associated Myasthenia Gravis. Curr Treat Options Neurol.

[13] Guptill JT, Sanders DB (2010). Update on muscle-specific tyrosine kinase antibody positive myasthenia gravis. Curr Opin Neurol.

[14] Hayashi M, Manabe K, Takaoka T (1995). Long-term change of anti-acetylcholine receptor antibody in patients with myasthenia gravis after thymectomy. Acta Paediatr Jpn.

